# Social preferences and psychopathy in a sample of male prisoners—a pilot study

**DOI:** 10.1038/s41598-024-59066-8

**Published:** 2024-04-09

**Authors:** Benjamin J. Kuper-Smith, Alexander Voulgaris, Peer Briken, Johannes Fuss, Christoph W. Korn

**Affiliations:** 1 Section Social Neuroscience, Department of General Psychiatry, Heidelberg University, Heidelberg, Germany; 2 Institute for Systems Neuroscience, University Medical Center Hamburg-Eppendorf, Hamburg, Germany; 3 Institute for Sex Research, Sexual Medicine and Forensic Psychiatry, University Medical Center Hamburg-Eppendorf, Hamburg, Germany; 4 Institute of Forensic Psychiatry and Sex Research, Center for Translational Neuro- and Behavioral Sciences, University of Duisburg-Essen, Essen, Germany

**Keywords:** Psychology, Human behaviour

## Abstract

Social decisions are influenced by a person’s social preferences. High psychopathy is defined by antisocial behaviour, but the relationship between psychopathy and social preferences remains unclear. In this study, we used a battery of economic games to study social decision-making and social preferences in relation to psychopathy in a sample of 35 male prison inmates, who were arrested for sexual and severe violent offenses (mean age = 39 years). We found no evidence for a relationship between social preferences (measured with the Dictator and Ultimatum Games, Social Value Orientation, and one-shot 2 × 2 games) and psychopathy (measured by the overall Hare Psychopathy Checklist-Revised score and both factors). These results are surprising but also difficult to interpret due to the small sample size. Our results contribute to the ongoing debate about psychopathy and social decision-making by providing crucial data that can be combined with future datasets to reach large sample sizes that can provide a more nuanced understanding about the relationship between psychopathy and social preferences.

## Introduction

Social interactions are a frequent and crucial aspect of everyday human life. By working together, humans can achieve things that cannot be done alone, but social interactions are often inherently ambiguous and complex, which can allow people to exploit others for their own gain.

Prosocial behaviour, defined as actions that benefit others, often at a personal cost, is influenced by a person’s personality^1^, such as Social Value Orientation^2^, which is a personal preference for how resources should be allocated between oneself and an anonymous other person that measures how (un)selfish a person is^3^. Personality traits such as psychopathy have also been shown to be associated with reduced cooperation rates^1^.

Psychopathy is a personality trait considered detrimental to social functioning and social cohesion. Although currently not officially recognised as a personality disorder by the Diagnostic and Statistical Manual of Mental Disorders^4^ or the International Classification of Diseases^5^, in some countries psychopathy is used in criminal justice settings as part of sentencing and parole decisions by judges and juries as a prognostic factor^6^. In a recent meta-analysis that considered the effect of personality traits on prosocial behaviour^1^, psychopathy was identified as one of the strongest negative correlates of prosocial behaviour. In addition to psychopathy, other established criminogenic needs such as impulsivity, ‘pro-crime’ attitudes and beliefs, relationships with criminal peers or poor problem solving skills^7,8^ may influence social decision-making and prosocial behaviour.

The relationship between psychopathy and social preferences is less clear: using the Ultimatum Game^9,10^, some studies find significant differences in decisions for people with high psychopathy compared to those with low psychopathy^11–13^, while others do not find any behavioural differences^14,15^, with other studies reporting mixed results^16–18^. For the Dictator Game^19–21^, similar disagreements exist for the relationship between psychopathy and behaviour in the Ultimatum Game^13,18,22,23^. To the best of our knowledge, Social Value Orientation^3^ has currently been investigated in only one study^18^, which found significant correlations between the other-score of the Ring measure Social Value Orientation score^24^ and psychopathy, but not for the self-measure. Overall, the evidence for a relationship between psychopathy and social preferences is mixed and requires further investigation. Additionally, many studies on psychopathy use only one or two tasks to measure economic preferences, which makes it difficult to compare the role of psychopathy between the tasks (e.g., how participants change their offers from the Dictator Game to the Ultimatum Game, and how this change might relate to psychopathy). We did not intend to study how different measures of economic preferences relate to each other.

In this study, we investigated whether there was a link between a person’s social preferences and their level of psychopathy, in a sample of male German prison inmates. Given the limitations of existing studies, we set out to describe the relationship between psychopathy and social preferences using a range of economic games applied to the same population. We had the opportunity to test 35 male inmates in a German prison, for whom Hare Psychopathy Checklist-Revised (PCL-R) scores were available. They completed a battery of economic tasks that assessed various aspects of their social preferences (Dictator Game, Ultimatum Game, and Social Value Orientation) and economic decision-making (2 × 2 games). We hypothesised that higher psychopathy would be associated with less generous social preferences and with reduced cooperation in the 2 × 2 games. We were particularly interested in whether the two factors that compose the overall PCL-R score have differential effects on social preferences.

## Methods

### Overall approach

This study represents an exploratory approach to study how psychopathy relates to social preferences. Specifically, we tested whether there was a negative relationship between psychopathy and prosocial preferences, such that the higher a person’s psychopathy score, the less money they would give to anonymous others in tasks that measure social preferences (Dictator Game, Ultimatum Game, Social-Value Orientation) and the less they would cooperate in social decisions (2 × 2 games). Due to the nature of the prison set-up, in which it was not possible to recruit further participants as one might in a standard laboratory study, there were some uncertainties before data collection (such as how many participants we might be able to recruit). For these reasons, we treat this study as an exploratory initial effort that we did not preregister.

### Ethics

All experiments were approved by the local ethics committee (Psychotherapeutenkammer Hamburg,

Germany, LPEK-0087) and the study was conducted in accordance with the good clinical practice guidelines as defined in the Declaration of Helsinki (2013). During recruitment and immediately before data collection, participants were informed that their individualised data would not be shared with anyone of the prison staff or the justice system, such that their responses in this study could not affect their chances for an early release on parole or have any other negative effect on their prison stay.

### Participants

The participants in our study were recruited in the social-therapeutic correctional facility in Hamburg, which belongs to the general prison system in Hamburg, Germany. Per German legislation, social therapy is considered the preferred form of correctional treatment in prisons for sex offenders who are sentenced for more than 2 years (German Federal Penal Execution Law §9). While this treatment is mandatory for sex offenders, non-sexual (mainly violent) offenders may also apply for it. A transfer back to the general prison facility is possible, if treatment goals or progress cannot be achieved due to reasons that lie in the person of the prisoner.

In Germany, Social Therapy Facilities (STFs) are located in prisons and provide treatment options to people who have committed considerable or repeated sexual and/or violent offenses^25^. Treatment in an STF is based on the RNR model aiming for risk reduction of reoffending and optimisation of resocialisation^8^. A combination of psychotherapeutic, educational, and occupational approaches is available with the goal of creating a therapeutic community inside the STF^26^. Specifically, in the STF in Hamburg, Germany, milieu therapy, individual therapy sessions, offense-specific group therapy, and strength-based approaches for sexual offender rehabilitation^27^, as well as general psychotherapy focussing on depression, substance abuse or trauma is offered to the inmates^28^. In a current study regarding treatment options in STFs in Germany, a general emphasis on cognitive-behavioural therapy with additional psychodynamic and schema therapy was identified^29^.

We recruited participants by visiting the prison about a week before the experiment. At each station (around 20 inmates each), we informed the group of inmates about the study, answered any questions, and handed out information sheets and consent sheets, which the inmates could read and fill in until the experiment. All inmates were invited to take part. Of the ≈ 80 inmates at the correctional facility, almost half (38) took part in our study. Of the 38 participants, 3 were excluded during data collection because their limited knowledge of German and English (the languages the experimenter BJKS could run the study in) meant the experimenter could not be certain that participants had fully understood the tasks. Of the remaining 35 participants, all completed the Dictator Game, Ultimatum Game, and Social Value Orientation task. 11 participants did not complete the 2 × 2 games, mainly due to time constraints (for further details see “2 × 2 games” Section. 2 × 2 games).

All participants were male and incarcerated for a sexual (14/35 = 40%) or severe violent offense (19/35 = 54%; of the remaining two participants, one was incarcerated for neither sexual nor violent crime, and for the other this information was not available). The mean age was 38.7 years (SD = 10.1).

The Psychopathy Checklist—Revised (PCL-R) is a psychological assessment tool developed by Robert D. Hare and currently the standard method for measuring and identifying psychopathic personality traits in offenders. It ideally requires a semi-structured interview by a trained mental health professional and the review of further information such as official prison and other judicial records, if available. If an interview is not possible, the PCL-R can be rated solely on the basis of file information. The PCL-R measure includes the individual criminal history and specific personality items (interpersonal, affective, lifestyle) and is regularly used in institutionalised individuals in prison or forensic psychiatric setting for diagnosis, treatment and prognosis planning. High psychopathy is generally associated with a poorer legal prognosis^30^. The scores were assessed independently from this study team by trained investigators independent from the prison system (for details of the procedure see^28,31,32^) weeks to months prior to our experiment. Thus, being able to test 35 participants with PCL-R scores presented a great opportunity for testing psychopathy with a high-quality psychopathy measure. Our study thus focussed on these 35 inmates for whom PCL-R scores were available.

For some participants, thorough information on some items of the PCL-R were missing, e.g. when available data in the file was sparse or when individuals did not fully cooperate during the interview (mean N of missing items per participant = 0.94; SD = 0.97, range = 0–3). In these cases, we used the adjusted PCL-R score and the adjusted scores for factor 1 (‘selfish, callous and remorseless use of others’) and factor 2 (‘chronically unstable, antisocial and socially deviant lifestyle ‘) according to the German version of the official manual^33^. In the following, whenever we refer to the overall PCL-R score or to either of the two factors, we refer to the adjusted score. Due to these missing items, we were unable to test the 4-factor model of psychopathy.

**Figure 1:**
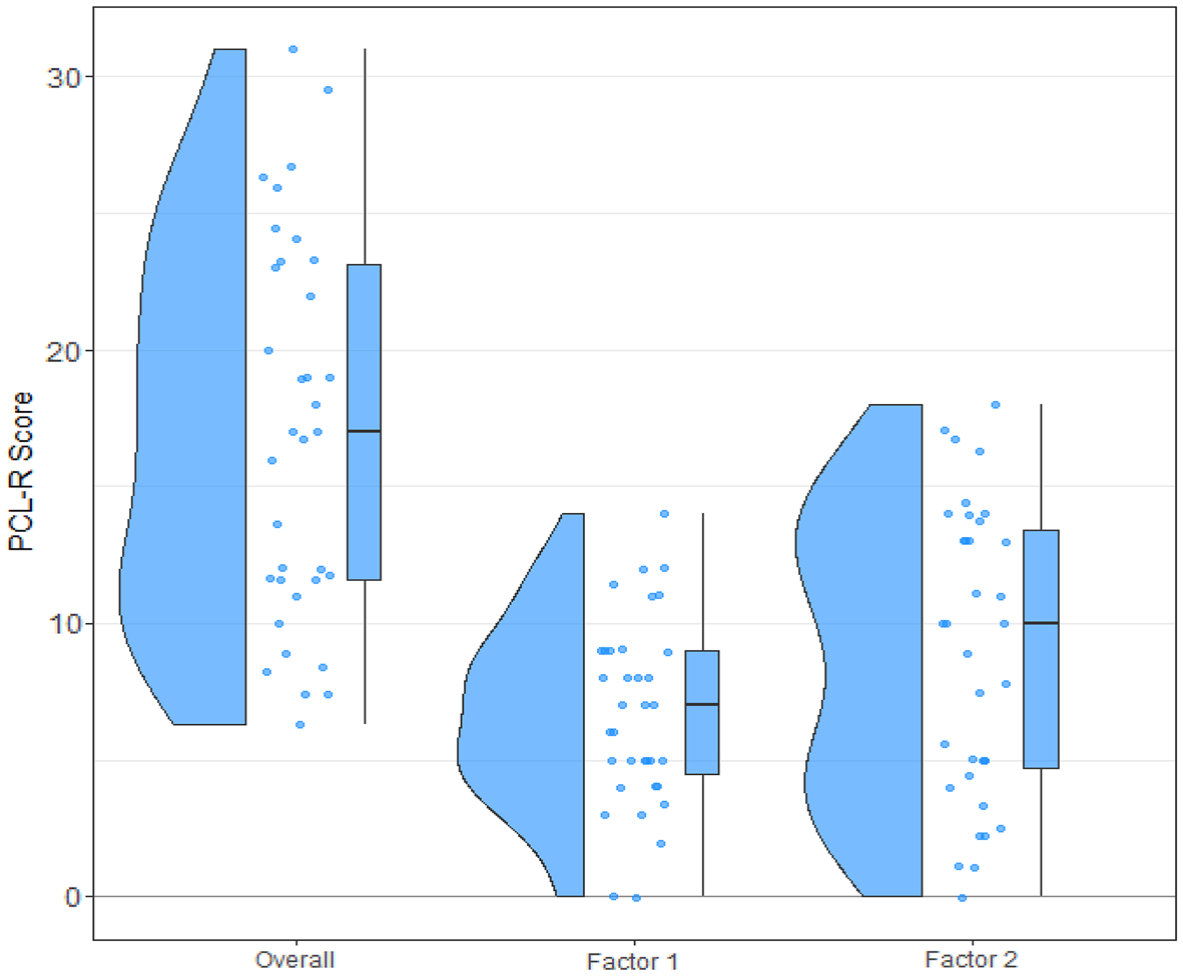
Descriptive of the participants’ PCL-R scores: overall, and each of the two factors. This raincloud plot^35^ includes individual data points, plus a box plot and a probability density function.

Our participants had a median PCL-R score of 17 (IQR 11.6–23.2), a median factor-1 score of 7 (IQR 4.3–9), and a median factor-2 score of 10 (IQR 4.6–13.6; see Fig. 1). One participant in our sample had a PCL-R score of at least 30 (the cut-off for psychopathy in the US) and an additional 4 participants had a PCL-R score of 25–29 (25 is the cut-off for psychopathy in Europe). Thus, only a small proportion (5/35) of our participants could be classified as having high psychopathy. Regarding (male) German-speaking prison populations, a study by Mokros et al.^34^ suggests a mean psychopathy score (PCL-R) of 18. A Wilcoxon Signed-Rank Test showed no significant difference between our sample and the median of 18 (Z = − 1.10, *p* = 0.27); the 16th and 84th percentile in our study (9 and 24.4) also closely match those reported in Mokros et al. (10 and 25). The PCL-R score ranges from 0 to 40, our lowest score is 6.3 and our highest score is 31. This means that our participants cover 62% of the entire possible range. Taken together, we conclude that our sample is representative of the (male) German-speaking prison populations.

The PCL-R scores were given anonymised to the experimenter after data collection was completed. The experimenter did not know anything about the participants other than their age and that they were inmates in the prison. We do not have further background information about the participants, such as potential confounders (such as antisociality).

### Procedure and tasks

The set of experiments reported here was part of a series of studies conducted on the same day with the same participants. The experiments reported here were the first in this series. Participants were brought in from their prison cells and completed the study with the experimenter. After finishing these studies, the participants completed further experiments, to be reported in other papers.

Participants completed several economic games^36^ (see Thielmann et al.^37^ for an overview of the differences between almost all tasks used in this study). All tasks focus on dyads: the Dictator Game and Social-Value Orientation are unilateral allocation tasks, the Ultimatum Game is a sequential allocation task, and the 2 × 2 games are simultaneous interdependent social dilemmas. The prison set-up made paying out different amounts of money to each participant difficult, so we instead kept the payoffs hypothetical and asked participant to imagine playing for these amounts of money. For the full set of original materials (in German), see Supplementary Material A.

In addition to the scientific reasons, the tasks were selected in part for practical purposes. There were about 15 min of testing time per participant for our study, such that the tasks had to be short and simple. We therefore selected a range of standard tasks in economic preferences and economic decision-making^37^ that cover allocation tasks (Dictator Game and Ultimatum Game), personal preferences (Social Value Orientation), and interdependent decision-making (2 × 2 games). We did not intend to study how different measures of economic preferences relate to each other.

All tasks in this study involve the participant allocating money or making decisions with financial consequences for themselves and for another person. The participants were told that this other person is anonymous and could be anyone from the population and that they would not know who this was—it could be anyone. The other person would not find out who had made the decisions, such that the situation was fully anonymous. In our initial description to the participants, we did not specify whether this other person was from within the prison or from outside of the prison (or any other prison), we merely stated that it could be anyone (“irgendjemand” in German). A few participants (< 5) specifically asked whether the other person was in prison or not, to which the experimenter responded that it’s not known who this person is and whether they were in prison or not, it could be anyone living in Germany at the time.

Before the experiment started, participants were reminded that the experiment was entirely separate from the prison administration and that whatever they said and did in these studies, no personally identifiable information would be given to their therapists, guards, judges, or anyone else, and that their data would not be individually identifiable. This was done to create a secure environment for the participants and ensure more open and honest answers, rather than saying what they thought would make them look good to the therapists.

#### Dictator game and ultimatum game

Participants completed a Dictator Game^20,21^ and an Ultimatum game^9,10^. For the Dictator Game, participants were told that they were given 100€ to split up between themselves and another person. The other person had no influence on how the money was allocated. The participant could keep all the money, give all to the other, or any combination in between. Participants then wrote down how much they would get and how much the other person would get. In all cases, participants correctly added up their values to 100€.

For the Ultimatum Game, participants were told that the situation was identical to before, but with one difference: now, the other person could reject the offer; if the offer was rejected, both players would get 0€, but if the offer was accepted both players would get what the participant wrote down. Again, participants wrote down how much they and how much the other person would get, and again all values added up correctly to 100€. Participants were then told that the roles were reversed and someone else had made offers, and the participant was now to decide whether to accept or reject those offers. The consequences of accepting and rejecting offers in the Ultimatum Game were repeated by the experimenter. Five different offers were made to the participants, always in the same order: self: 50 other 50 (50/50); 20/80; 30/70; 10/90; 0/100. Participants were told that these were 5 different hypothetical people who made the offers, and that they (the participants) should decide anew for each offer. Participants then circled whether they accepted or rejected each offer, and participants indicated how fair they thought the offer was, using any number from 0 (totally unfair) to 100 (totally fair).

#### Social value orientation

Social Value Orientation (SVO) describes a person’s preferences about how money should be allocated between themselves and another person. Participants completed a visually simplified version of the paper-version of a commonly used task^38^ that measures Social Value Orientation across 15 items (6 items measure their primary SVO score and 9 items measure their secondary prosocial motivation score). For each item, participants were shown a continuum of joint payoffs for themselves and another person and selected the allocation option that appealed most to them. The measure we used in this study provides a degree of prosociality for each participant (with a higher degree indicating a more prosocial SVO), which can then be categorised into one of four types (altruistic, prosocial, individualistic, and competitive), and for the prosocials, it also provides a prosocial motivation score (to what degree they minimised inequality or maximised joint payoff). The Social Value Orientation slider-measure can be analysed only if a participant’s responses are transitive (e.g., if one prefers A over B and B over C, one should prefer A over C), so any participant whose responses are not transitive are excluded (for details, see Murphy et al.^38^).

#### 2 × 2 games

Participants also played four different 2 × 2 games^39^: Prisoner’s Dilemma, Stag-Hunt, Hawk-Dove, and No Conflict. 2 × 2 games are economic games in which 2 players choose simultaneously between 2 options, leading to 4 possible outcomes (both cooperate (CC), both defect (DD), player 1 cooperates and player 2 defects (CD), and the reverse (player 1 defects and player 2 cooperates; DC); see Table 1):

**Table 1:** The logic of 2 × 2 games as displayed from player 1’s perspective.

Player 1/Player 2	Cooperate	Defect
Cooperate	CC	CD
Defect	DC	DD

The outcomes depend on both players’ decisions. In this study, we used 4 different games (see Table 2). These 4 games are identical apart from the ordering of payoffs, which consists of swapping two payoffs to get from one to the next:

**Table 2:** The four different games used in this study. The different games are defined by the order of the payoffs as displayed here from player 1’s perspective.

	Best outcome	Second best outcome	Second worst outcome	Worst outcome
Prisoner’s Dilemma	DC	CC	DD	CD
Hawk–Dove	DC	CC	CD	DD
Stag-Hunt	CC	DC	DD	CD
No Conflict	CC	DC	CD	DD

**Figure 2:**
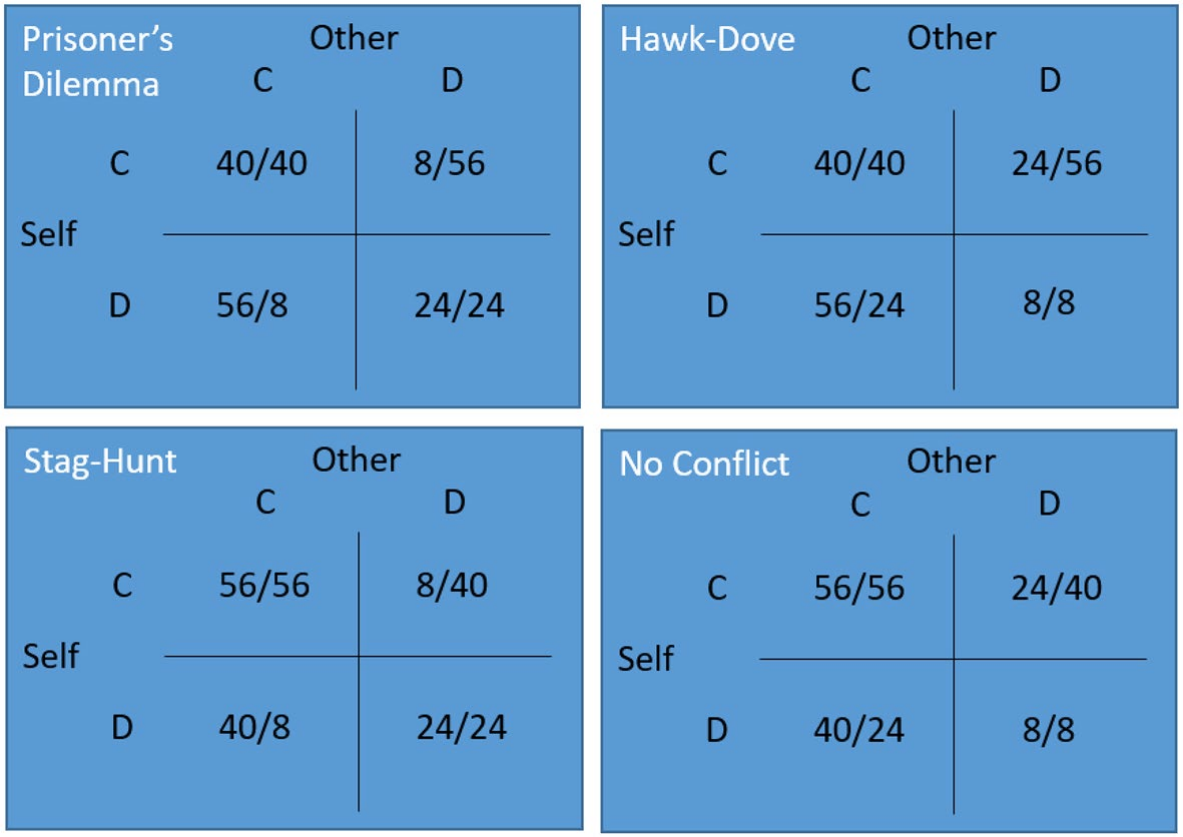
The payoffs used in this study for the 2 × 2 games. The number to the left of the slash indicates the payoff for Self, while the number to the right of the slash indicates the payoffs for the other player.

Depending on the ordering of the outcomes, different games emerge with different factors being relevant for deciding between the two options. The Prisoner’s Dilemma^40^ (DC > DD > DD > CD) presents a dilemma between what’s best for the individual and what’s best for the 2 players combined: no matter what the other player does, one gets a higher payoff by choosing D—but if both players choose C, they get the highest joint outcome. Hawk-Dove^41,42^ (also known as Chicken or Snowdrift) has an identical structure to the Prisoner’s Dilemma with one change: getting exploited is no longer the worst option, mutual defection is; Hawk-Dove has been used to model situations in which mutual destruction is the worst possible outcome, such as in nuclear wafare^43^. Stag-Hunt is also identical in structure to the Prisoner’s Dilemma, but with the difference that in Stag-Hunt mutual cooperation is the best possible individual outcome (rather than exploiting the other player); Stag-Hunt has been used to model hunting and foraging situations^44^. No Conflict is a game with no real conflict: mutual cooperation is the best possible outcome and mutual defection is the worst possible outcome; it can be used to identify competitive people (who would defect to gain more points than the other). For the payoff matrices used in this study, see Fig. 2. All 2 × 2 games in our study were symmetric, such that both players had the same options with the same outcomes. All games were presented in matrix form (see^45^ for a distinction between matrix form and decomposed form). We did not label the options ‘cooperate’ or ‘defect’, but labelled them option ‘A’ and option ‘B’. As with the other tasks, the ‘other’ player was a hypothetical other person who did not actually make any decisions.

The order of the four 2 × 2 games was randomized and the first game was used to explain the general set-up of 2 × 2 games. To test whether participants understood the instructions, the experimenter asked a few basic questions (such as ‘if you choose C and the other chooses D, how much money does the other person get?’). If players got the question wrong, the task was explained again. If participants did not manage to answer such questions correctly, despite several different explanations and answering questions, the task was stopped and participants moved on to the next task. If participants answered the questions correctly, they then decided between the two options for each game. It was clarified that each game had a slightly different order of payoffs and that participants should carefully check again what the consequences of their actions would be. After deciding what to do in each game, participants also estimated what they thought the other player would do. Participants estimated what the other person would do, using a scale from 0 (definitely cooperate) to 100 (definitely defect) with 50 as an explicit anchor if they had no idea what the other would do. For all 2 × 2 games, it was made clear that participants should decide anew each time; it was no problem if they gave the same response each time, or if they gave different responses each time, all that mattered was that they thought about each question as if the other questions did not exist. Once all 2 × 2 games and expectations were completed, participants described how good it would be for them to get the money from the 4 values presented in the 2 × 2 games right now. In other words, we asked them to rate from 0 (strong dislike) to 100 (strong like) how good it would be for them if they were to get 8/24/40/56€, independent from the interdependent decision-making inherent in 2 × 2 games.

### Analyses

For all tasks, we test whether participants’ behaviour in these tasks is related to their PCL-R scores, always for their overall score as well as their scores for the first and second factors. For offers made in the Dictator Game, Ultimatum Game and the difference between these two, we correlate participants’ offers with their PCL-R scores. For the offers received in the Ultimatum Game, we correlated the lowest offers accepted with the PCL-R scores. For Social-Value Orientation, we correlated overall Social-Value Orientation angles, as well as their normalised distance from the ideal inequity aversion options with PCL-R scores. For 2 × 2 games, we correlated overall cooperation rates with PCL-R scores. All correlations are Spearman correlations due to the non-normal distributions.

## Results

### Dictator game and ultimatum game

#### Make offers

Participants played a Dictator Game followed by an Ultimatum Game. In the Dictator Game, the participants decided how to split the money. In the Ultimatum Game, participants acted as both the proposer and the receiver: they made an offer, and had to rate different offers and decide whether to accept or reject them.

The median offer was 50/50 (€ self/€ other) in both the Dictator Game and in the Ultimatum Game. Many participants offered equal amounts to themselves and the other person in the Dictator Game (60%) and in the Ultimatum Game (60%; although the proportion is the same in both games, not all of these are the same people). 60% of participants gave the same amount in both games. Of the remaining 40%, all but one participant (37.14%) gave the other person more money in the Ultimatum Game than in the Dictator Game, which is to be expected^46^.

As Table 3 shows, there were no significant correlations (Spearman due to non-normal distributions) between offers made in the Dictator Game, offers made in the Ultimatum Game, or the difference thereof, with either the overall PCL-R score, or the scores of the two factors, even without correcting for multiple comparisons. Given our small sample size of 35 participants, after analysing our data, we ran a sensitivity analysis with G*Power, which showed that with an α = 0.05 and a β = 0.8 a correlation would have to be at least 0.4 for significant results; the largest correlation of − 0.26 (offers made in the Dictator Game and Factor 1) would have required a sample size of 90 participants (with the same α and β) to yield significant results. Generally, the effect sizes of these analyses are quite small: for the overall PCL-R score, the largest correlation is r = − 0.12 (for offers made in the Dictator Game).

**Table 3:** Correlations between offers made in the Dictator Game, the Ultimatum Game and their difference with the overall PCL-R score and the two factors.

	PCL-R overall		Factor 1		Factor 2	
	r	*p*	r	*p*	r	*p*
Dictator offers made	− 0.1733	0.3195	− 0.1804	0.2998	− 0.1540	0.3769
Ultimatum offers made	− 0.1632	0.3489	− 0.2862	0.0955	− 0.0651	0.7103
Difference between Dictator and ultimatum offers	− 0.0640	0.7149	0.0000	0.9967	− 0.0726	0.6785

#### Receive offers

In the next part, participants were given offers by an anonymous other person and had to decide whether to accept them, and had to rate the offers for how fair the participant thought the offer was.

**Figure 3:**
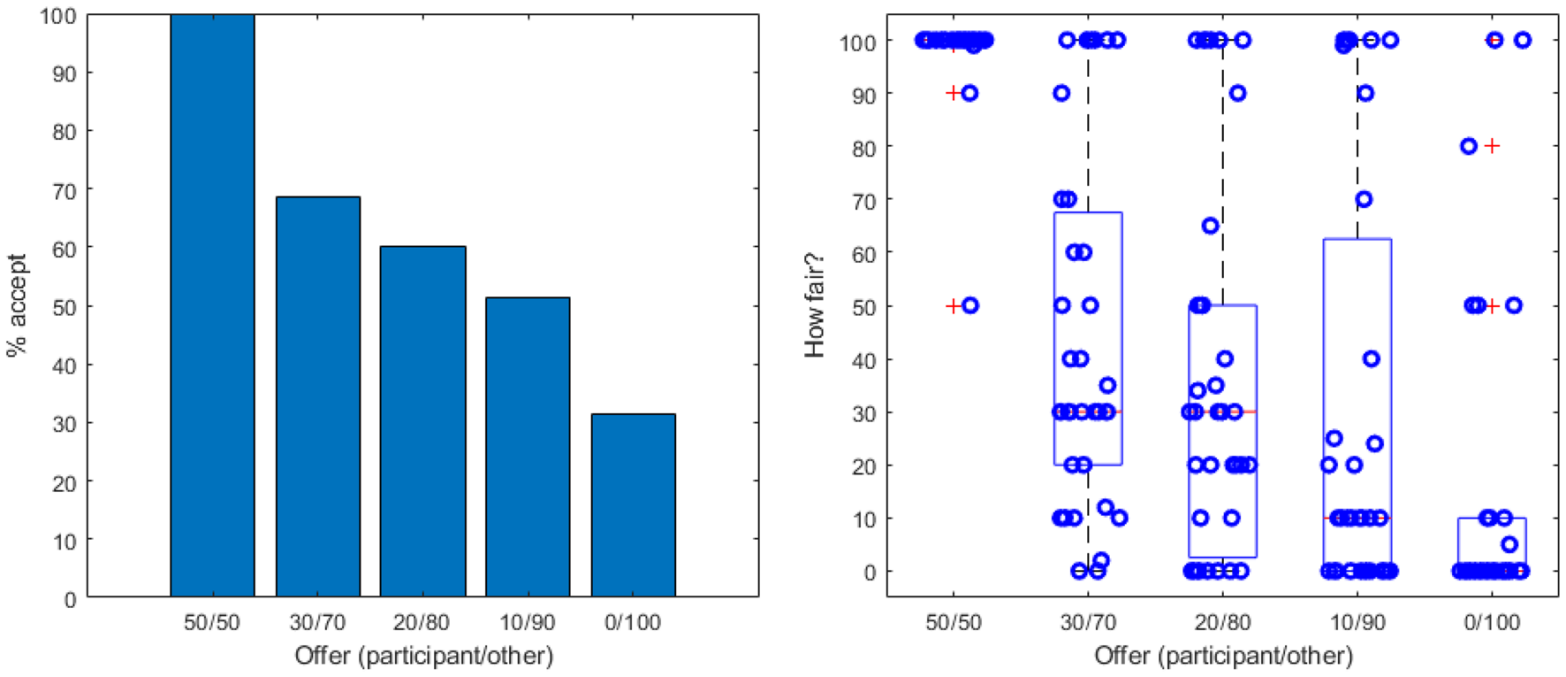
Acceptance percentage (left) and fairness ratings (right) for the different offers participants received in the Ultimatum Game. ‘Offer (participant/other)’ means that the left value (e.g., 30) was offered to the participant and that the right value (e.g., 70) the other player offered themselves. All offers in this game thus either split the money in half or gave the participant less than the other player. For the fairness ratings, the scale ranged from 0 (not at all fair) to 100 (totally fair).

All participants accepted a 50/50 split and almost all (32/35) rated it as 100 out of a 100 for fairness (see Fig. 3). For the remaining offers, both the amount of offers accepted and the fairness ratings were reduced substantially, such that for a 0/100 split only 31.4% participants accepted the offer and the mean fairness rating was 16.1 (out of 100). Eight participants still accepted this lowest rating and rated its fairness as more than 0: this was not an error, but a deliberate decision by the participants. When the experimenter asked the two participants who had rated the 0/100 offer as perfectly fair (100 out of 100) after the experiment had finished why they had done so, the participants responded that it was the other person’s money and they could do whatever they wanted with it; it was fair to not share your own money. Nonetheless, most participants found the 0/100 split extremely unfair: median and mode fairness rating of the 0/100 split were both 0.

**Figure 4:**
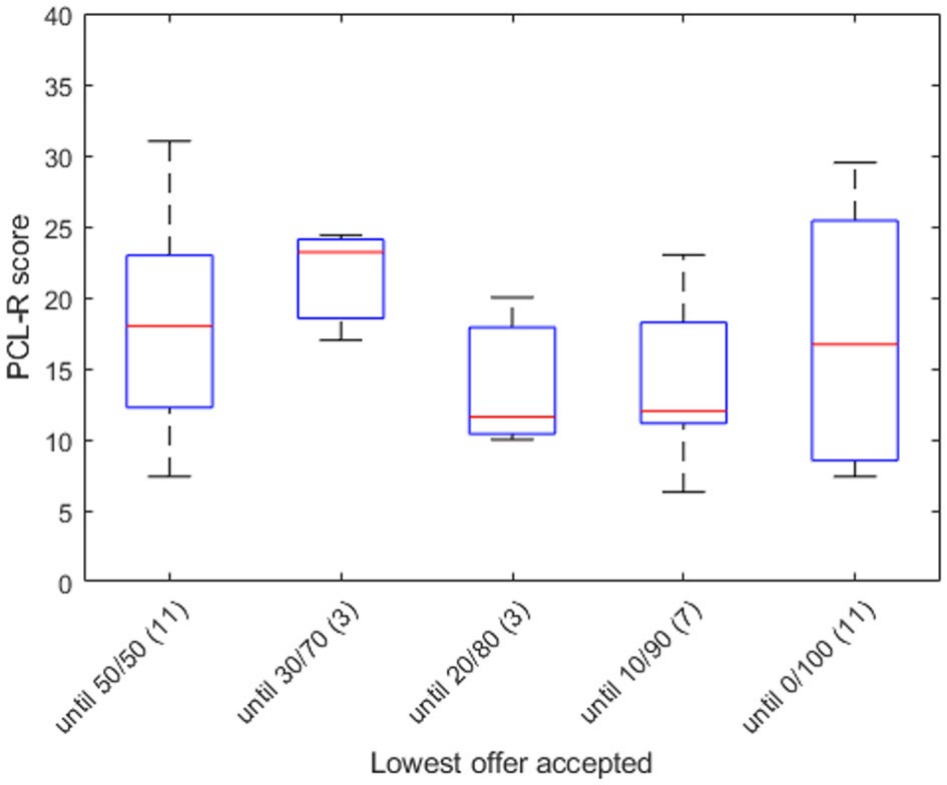
The relationship between PCL-R score and the lowest offer participants accepted (N of participants in each category in parentheses).

To test whether accepting offers in the Ultimatum Game correlated with psychopathy, we classified participants into groups depending on which was the lowest offer they accepted. For example, someone who accepted the 50/50 offer and the 30/70 offer but rejected all other offers was classified as ‘30/70 lowest offer accepted’ (this analysis was possible because all participants were transitive/consistent with respect to accepting offers). The number of participants in each group was too small for statistically meaningful comparisons between the groups (e.g., only 3 participants accepted until 30/70 and only 3 people accepted until 20/80), but this approach provided a range of responses which we could use to run Spearman correlations. There were no significant correlations between people’s lowest offer accepted and their overall PCL-R score (r = − 0.1414, *p* = 0.4177), for the first factor (r = − 0.0500, *p* = 0.7756), or the second factor (r = − 0.1885, *p* = 0.2781), again before correcting for multiple comparisons (see Fig. 4). Again, our small sample size limits the interpretability of our results.

In summary, there was no evidence that psychopathy had an effect on offers made, rejected, or evaluated for fairness in our sample, neither for the overall PCL-R score, nor for the two factors.

### Social value orientation

6 of our 35 participants were excluded from this section due to their non-transitive data (see Methods).

The vast majority of our participants (86%; 25 out of 29) were categorised as prosocial. Only 3 of our participants (10%) were categorised as individualists, and only 1 person as competitive (4%), with no altruists. Of the prosocials, 18 (72%) were inequity minimisers, 3 (12%) were joint-gain maximisers, and 4 (16%) made intransitive choices for the secondary items and could not be classified.

There were no significant correlations between the participants’ overall SVO-angle and their overall PCL-R score (r = 0.2116, *p* = 0.2706), their scores for the first factor (r = 0.0979, *p* = 0.6133) or the second factor (r = 0.1401, *p* = 0.4684) of the PCL-R measure. There were not enough responses to compare participants from the different categories (i.e., 1 competitive vs 3 individualists vs 25 prosocials vs 0 altruists); similarly, for the prosocial subcategories there were not enough joint-gain maximisers (3) to compare them to inequity minimisers. To further analyse the secondary items, we therefore instead correlated the participants’ PCL-R score with their normalised distance from the ideal inequity aversion options with the psychopathy measures, again with no significant correlations (overall: r = − 0.1849, *p* = 0.3984; factor1: r = 0.0340, *p* = 0.8775; factor 2: r = − 0.3375, *p* = 0.1153). The previously mentioned limitations of our sample size are exacerbated here by having to exclude 6 further participants.

### 2 × 2 games

Of the 35 participants who took part, 11 did not take part in the 2 × 2 games. This was mainly due to the strict time schedule imposed by the experimenters that left little room for delays, and not due to any inherent differences between the participants who participated in the entire study and those who did not take part in the 2 × 2 games. The 24 participants who completed the entire study did not differ from the 11 who did all tasks apart from the 2 × 2 games with respect to overall PCL-R score (median_24_ = 17.95, mean_11_ = 16.00; t_Welch_(17.61) = 0.12, *p* = 0.90), factor 1 (mean_24_ = 7.70, mean_11_ = 5.00; t_Welch_(13.15) = 0.83, *p* = 0.42) or factor 2 (mean_24_ = 8.90, mean_11_ = 10.00; t_Welch_(21.60) = 0.05, *p* = 0.96). There was also no evidence that the two groups differed with respect to their age (mean_24_ = 38.1; mean = 39.9; t_Welch_(21.49) = − 0.50, *p* = 0.62). We thus conclude that for the purpose of our analysis there are no important differences between the two groups and will use the smaller sample (24 participants) for this section.

**Figure 5:**
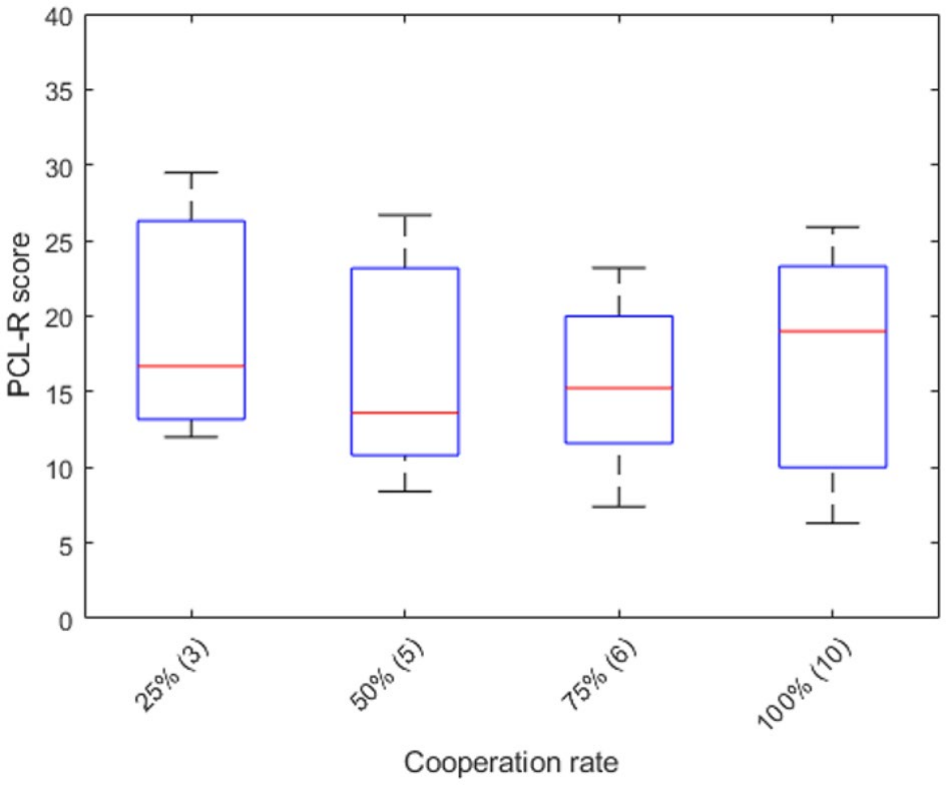
The effect of PCL-R score on cooperation rate, averaged per participant across the four 2 × 2 games (N of participants in each category in parentheses).

There were no overall differences for either cooperation rate (χ^2^(3) = 2.23, *p* = 0.53) or expected cooperation from the other person (F(3, 92) = 1.29, *p* = 0.2823; see Table 4 for mean values) between the four 2 × 2 games. There were no correlations (Spearman) between average cooperation rate per person and their overall PCL-R scores (r = − 0.0050, *p* = 0.9814; see Fig. 5), their factor 1 scores (r = − 0.1838, *p* = 0.3899), or their factor 2 score (r = 0.0505, *p* = 0.8149). Due to the relatively small sample size and dichotomous nature of the responses, potential comparisons in PCL-R scores between those who cooperated and those who defected in each of the individual games was not possible (the number of people who defected ranged from 3 to 7 in each of the games). Due to this reduced sample size, the previous limitations in interpretability apply even more to the results of this section.

**Table 4:** Cooperation rate and expected cooperation rate across the 4 games.

	Cooperation rate (%)	Expectation other person cooperation (%)
Prisoner’s dilemma	71	46
Stag-Hunt	67	54
Hawk–Dove	83	58
No conflict	75	62

## Discussion

In our sample of male prison inmates, we found no evidence that social preferences were related to psychopathy, neither for overall psychopathy as measured with the PCL-R measure, nor for the two factors of the PCL-R measure. Due to the small sample size, it is difficult to tell whether these results represent true null-effects or whether they are the consequence of an underpowered study. In either case, given how difficult it is to collect data from prison inmates who have PCL-R scores (at least in Germany), the best way forward might be to collect and publish smaller individual studies that can be combined into a future meta-analysis.

Compared to previous studies, our participants were surprisingly generous. A recent meta-analysis^46^ of studies using Dictator Games and Ultimatum Games found that people offer the other person on average 25% of the money in the Dictator Game and 42% in the Ultimatum Game. In our study, participants offered the other person on average 41% of the money in the Dictator Game and 50% in the Ultimatum Game, thus offering the other person much more than other studies typically find. Similarly, in our study participants have higher Social Value Orientation scores than in most studies: e.g., all studies mentioned in a meta-analysis^2^ have a prosocial rate of less than the 86% in our sample (although these studies appeared before the improved slider measure used in our study came out), and the results from the original study that introduced the slider measure^38^ and from a recent study that included several different samples^47^ also suggest that typical samples show lower rates of prosocial people. The prosocial participants in our sample are predominantly inequality averse, which matches the results from a recent study^47^. Thus, our participants seem to be more prosocial than participants in most other studies, but with similar prosocial motivation.

Multiple potential reasons may explain why the participants in our sample are more generous and prosocial than in other studies. First, it is possible that participants acted nicer than usual because they were worried that their data would be shared with prison staff or therapists that are responsible for parole decisions. This is particularly likely for studies like ours where it is fairly obvious which responses are considered unfair or socially disruptive, and that thus might receive disapproval if the data were to be shared. The experimenter explained to each participant before the experiment started that any data from the experiment would be completely confidential and not shared with anyone at the prison or who could affect their parole decision, but it is possible that such concerns nonetheless affected the participants’ decisions. Second, it is possible that the participants of this study acted more generously than they might usually do because they wanted to appear generous to the experimenter. In most studies participants’ responses are not immediately shared with the experimenter (e.g., filling in a survey online from home, or in a lab into a computer with the experimenter in another room), but in this study the experimenter sat at a table with the participants. This was done for practical reasons (there was not a lot of time for delays, and it was quicker this way to explain the study and ensure that participants had understood everything). Thus, acting selfishly in this setting meant also implicitly telling another person that they were doing so. It is thus likely that this too increased the proportion or cooperative responses. Third, this study was conducted with inmates at a social-therapeutic correctional facility, where the inmates take part in therapy and are encouraged to act in a more prosocial manner in general. This too might have led to higher overall prosociality in our participants. Fourth, our experiment was hypothetical rather than incentivised. Although a meta-analysis found no overall effect of incentivisation^1^, we cannot exclude that the hypothetical set-up in combination with the aforementioned other potential reasons lead to higher prosociality in our participants.

The aim of this study was to assess the relationship between social preferences and psychopathy. The unusually kind social preferences are interesting and could interfere with assessing the relationship between social preferences and psychopathy, if there is an interaction between social preferences and psychopathy (such that e.g., the people with high psychopathy became disproportionally more generous than people with low psychopathy), or if ceiling effects preclude us from running the planned analyses. There is no evidence in our study for such an interaction between psychopathy and generosity, and while there were some ceiling effects that precluded us from running some analyses, this was mainly due to our small sample size, rather than necessarily due to high cooperation rates (e.g., in the 2 × 2 games, there were not enough participants who defected to compare the psychopathy scores of those who cooperated with those who defected, but with a sample size of 24 participants, splitting the sample into two groups was always going to be difficult). Thus, although the participants in our sample were unusually generous, this was not a direct problem for the analysis of the data or the interpretation of the results.

A further question arises from the null findings in the 2 × 2 games. In a recent meta-analysis^1^, psychopathy was identified as having a strong correlation with reduced prosocial behaviour. In our study, however, there was no significant correlation between cooperation rates and psychopathy scores (overall PCL-R scores and each of the two factors). A surprising result in our study is that the participants did not show higher cooperation rates for No Conflict than for any of the other games. This is surprising given that No Conflict is named so because this game incentivises players to cooperate: mutual cooperation leads to the highest possible payoff and mutual defection leads to the lowest possible payoff. No Conflict can thus almost be seen as a control condition: if participants don’t cooperate in No Conflict, it is possible they did not pay particularly close attention to the precise payoff structure of the game. In our study, 25% of participants did not choose cooperation in No Conflict. In our study, the order of the four 2 × 2 games was randomised and the experimenter used the first game to explain the task to the participants. Although the experimenter did ensure that each participant fully understood the first game they played and although the experimenter did highlight to the participants that the subsequent games would feature a slightly different payoff structure that the participants should study carefully before deciding, it is possible that a substantial proportion of our participants did not fully reconsider the payoff structure of the following games, thus leading to null findings. This potential problem is unique to the 2 × 2 games and should not apply to any of the other tasks in our study.

Future studies could seek more nuanced experimental designs than the ones used here, and try to establish the ecological validity of the tasks used here by examining the relationship between the specific tasks used in this study and the real-world socially disruptive behaviour that defines psychopathy in violent or sexual offenders. We used standard tasks for eliciting economic preferences in a contextless and generic situation: the other person is anonymous and the money to be allocated was a windfall. It was noticeable that a substantial proportion of participants in this study asked questions to specify the context in which the two people and the money were: who was the other person, where did the money come from, did they work together, did they do equal amounts of work and effort etc.? While in other studies the experimenter was also asked such questions by the participants, it seemed particularly prevalent in this study. While this is anecdotal, the question remains: if psychopathy is in part defined by abnormal social relationships, then why do our results not indicate any effects? While there are multiple possible reasons for this, one currently largely untested possibility is that the contextless paradigms used here are not nuanced enough to elicit the kinds of situations in which people with high psychopathy show abnormal behaviour (e.g., two people both contributed equally to a project but the other person did not share evenly). Future studies seeking to study the relationship between psychopathy and social preferences could profit from using more nuanced designs, such as iterated 2 × 2 games against players with different strategies, (asymmetric) decomposed games^45^, or allocation tasks where the money had to be earned by the two players prior to the allocation decision. Additionally, due to the prison set-up, in our study the participants’ responses had to be hypothetical—if possible, future studies should seek to incentivise decisions. Furthermore, our current approach assumes a linear relationship between psychopathy and antisocial behaviour—with more data, future studies could test non-linear relationships (e.g., psychopathy reduces prosocial behaviour only above the clinical cut-offs). Finally, there are several confounders (e.g., impulsivity, antisociality, or duration of therapy) that we did not control for; if possible, future studies could also seek to more comprehensively capture and control for such additional factors.

In line with the previously mixed results on psychopathy and social preferences, we found no evidence for any statistically significant relationships between psychopathy and social preferences. Interpreting our results is limited due to the small sample size. The number of participants in our study was limited by the fact that we recruited in a specific prison. Recruitment across multiple prisons is challenging but would be desirable to obtain larger sample sizes. Alternatively, we suggest publishing smaller individual studies with full transparency (including sharing the data), so that future meta-analyses can combine the data from such individually potentially underpowered studies into meta-analyses that achieve the statistical power required to detect even small effect sizes.

### Supplementary Information

Supplementary Information.

## Data Availability

All code used for the analysis of this project is publically available at https://github.com/dnhi-lab/social_prison. All data generated or analysed during this study are included in this published article, with one exception: to protect our participants’ anonymity, for the PCL-R scores (which are based in part on a person’s criminal history and might make people individually identifiable especially for high scores) we only display the overall category from 1 (very low: PCL-R = 0–8) to 5 (very high: PCL-R = 33–40). For access to the full dataset, please contact Christoph Korn.
