# Supplementary material for: Social preferences and psychopathy in a sample of male prisoners—a pilot study
Source: Sci Rep. 2024 Apr 9;14:8344. doi: 10.1038/s41598-024-59066-8 (PMC11004005; doi:10.1038/s41598-024-59066-8)
Supplement: Supplementary file 1 — Supplementary Information. [file 41598_2024_59066_MOESM1_ESM.pdf]

Supplementary Materials

A: Full materials.

Please note that the written version presented here was used to explain the task to the participants verbally. This was done because many participants preferred a verbal explanation, and because several participants did not speak German (particularly well), such that the experimenter had to simplify the language occasionally.

SVO:

For the Slider questions, see Murphy et al (2011). These were presented first, randomly either version A or version B. The original materials were somewhat confusing, so we simplified the layout and explained the task verbally (rather than written instructions as in the original study).

2*2:

The task was explained by the experimenter to each participant, using whatever 2*2 game payoff matrix appeared first. Each of the 2x2 games was presented on a separate sheet of paper (here we only show one as an example).

“

Da Sie jetzt das Experiment verstehen, kommen wir jetzt zu Ihren Entscheidungen.

Hier sind die möglichen Kombinationen:

	Die andere Person entscheidet sich für A	Die andere Person entscheidet sich für B
Sie entscheiden sich für A	Sie: 40€, andere: 40€	Sie: 8€, andere: 56€
Sie entscheiden sich für B	Sie: 56€, andere: 8€	Sie: 24€, andere: 24€

Bitte entscheiden Sie sich jetzt.

Ich wähle:

☐ **A** ☐ **B**

”

Participants were also asked for their expectation of the other person. For this, participants wrote their number from 0 to 100 below their decision.

“

In den vorherigen Entscheidungen ging es um verschiedene Geldbeträge, die Sie und eine andere Person bekommen konnten. Jetzt interessiert uns wie gut oder schlecht es für Sie persönlich wäre diese Geldbeträge zu bekommen.

Im Folgenden können Sie für jeden Geldbetrag eine Zahl auswählen, die angibt, wie sehr Ihnen der Betrag gefällt.

Bitte wählen Sie eine Zahl von 0 bis 100. 0 bedeutet, dass Ihnen der Geldbetrag überhaupt nicht gefällt. 100 bedeutet, dass Ihnen der Geldbetrag hervorragend gefällt. Sie können irgendeine Zahl zwischen 0 und 100 auswählen; je größer die Zahl, desto besser gefällt Ihnen der Betrag.

8€:

24€:

40€:

56€:

“

DG & UG:

DG:

“

In diesem Teil der Studie werden Sie wieder hypothetische Entscheidungen um Geldbeträge fällen.

Die Situation ist folgende: Ihnen und einer anderen Person werden 100€ gegeben, aber nur Sie können darüber entscheiden wie das Geld aufgeteilt wird. Die andere Person hat keinen Einfluss darauf, wie das Geld aufgeteilt wird und wird auch nicht wissen, dass Sie die Entscheidung gemacht haben.

Sie können die 100€ so aufteilen wie Sie möchten.

Bitte geben Sie an wieviel Geld von den 100€ Sie selbst behalten und wie viel der andere bekommt:

Sie bekommen:

Die andere Person bekommt:

“

UG make offer:

“

Dieser Teil ist sehr ähnlich wie der vorherige Teil: wieder können Sie Geld unter sich selbst und einer anderen Person aufteilen. Alles ist wie vorher, aber es gibt einen Unterschied: die andere Person kann Ihre Geldverteilung ablehnen. Wenn die andere Person mit Ihrer Geldverteilung nicht zufrieden ist, kann sie die Verteilung ablehnen und dann bekommt keiner Geld. Wenn die andere Person die Geldverteilung annimmt, bleibt es bei der Verteilung, die Sie vorgeschlagen haben.

Wieder können Sie das Geld so verteilen wie Sie möchten:

Sie bekommen:

Die andere Person bekommt:

”

UG receive offers:

“

Stellen Sie sich jetzt vor, dass eine andere Person die Geldverteilung vorschlägt und dass Sie entscheiden müssen, ob Sie die Verteilung annehmen.

Stellen Sie sich vor, 5 verschiedene Personen haben folgende Verteilungen vorschlagen. In jedem Fall müssen Sie entscheiden, ob Sie die Verteilung annehmen oder ablehnen. Bitte umkreisen Sie Ihre Antwort:

Sie bekommen: 50€

Andere Person bekommt: 50€

Ich nehme die Verteilung an Ich lehne die Verteilung ab

Sie bekommen: 20€

Andere Person bekommt: 80€

Ich nehme die Verteilung an Ich lehne die Verteilung ab

Sie bekommen: 30€

Andere Person bekommt: 70€

Ich nehme die Verteilung an Ich lehne die Verteilung ab

Sie bekommen: 10€

Andere Person bekommt: 90€

Ich nehme die Verteilung an Ich lehne die Verteilung ab

Sie bekommen: 0€

Andere Person bekommt: 100€

Ich nehme die Verteilung an Ich lehne die Verteilung ab

”

End of materials

B: Contribution table

	BJ Kuper-Smith	A Voulgaris	P Briken	J Fuss	CW Korn
**Conceptualization**	3	0	0	0	3
**Data analysis**	3	0	0	0	1
**Data collection**	3	1	0	0	0
**PCL-R database**	0	0	3	0	0
**Prison liaising and organisation**	0	2	0	3	0
**Supervision**	0	0	0	0	3
**Writing: original draft**	3	0	0	0	0
**Writing: review & editing**	3	2	1	2	3

Figure 1 - The authors’ contributions for this paper. Higher numbers (and darker colours) indicate more contribution: 0 (no contribution), 1 (supporting contributions), 2 (medium contributions), and 3 (lead contributions)
